# Supplementary material for: The interactome of intact mitochondria by cross-linking mass spectrometry provides evidence for coexisting respiratory supercomplexes
Source: Mol Cell Proteomics. 2017 Dec 8;17(2):216–32. doi: 10.1074/mcp.RA117.000470 (PMC5795388; doi:10.1074/mcp.RA117.000470)
Supplement: Supplemental Data [file supp_RA117.000470_133922_0_supp_23978_fzffwf.zip › spectra_annotation/mito_spectra_annotation/221-1-11-1-11-1.pdf]

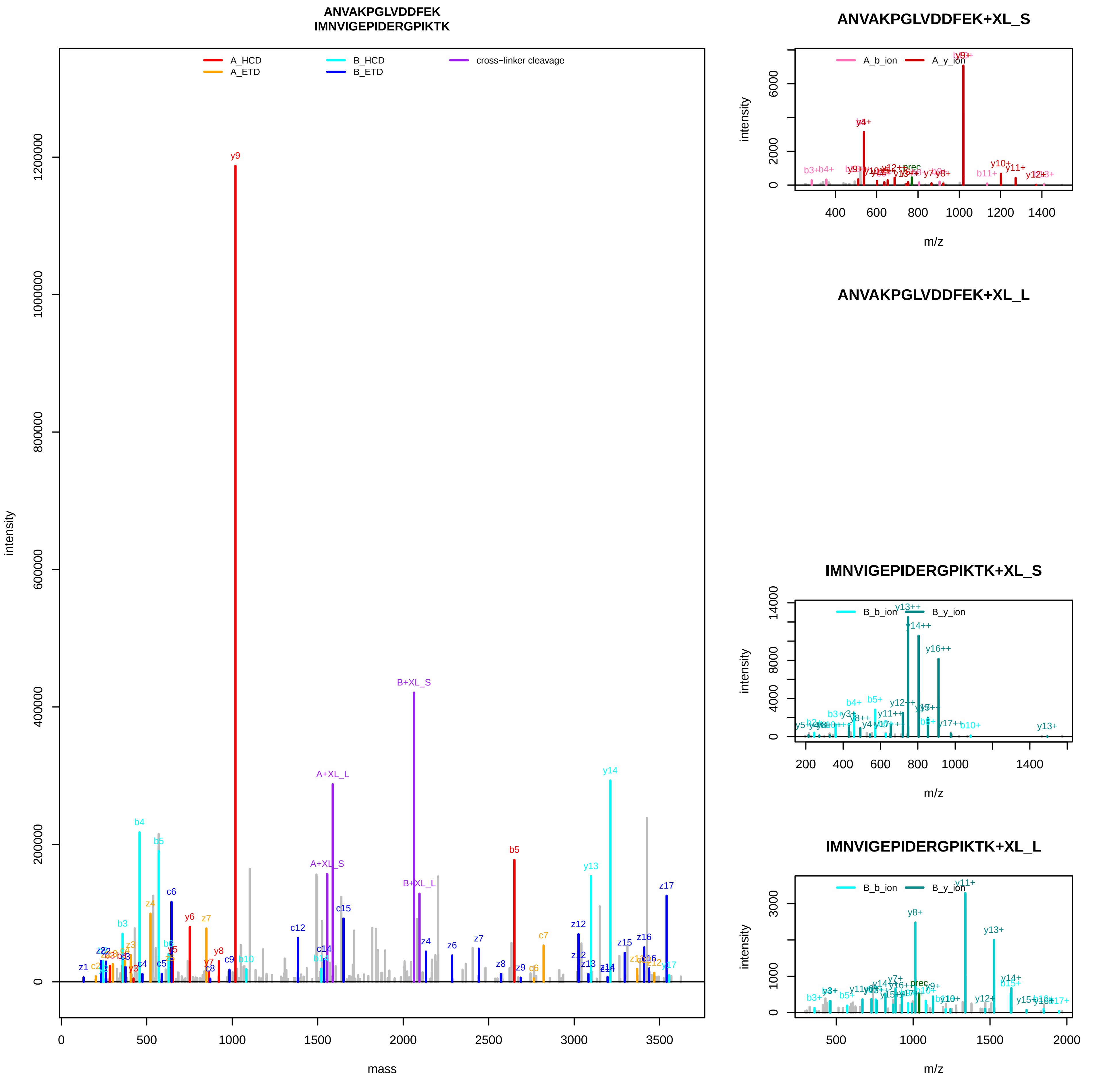

ANVAKPGLVDDFEK
IMNVIGEPIDERGPIKTK
A_HCD
A_ETD
B_HCD
B_ETD
cross−linker cleavage
intensity
mass
0
200000
400000
600000
800000
1000000
1200000
500
1000
1500
2000
2500
3000
3500
y9
B+XL_S
A+XL_L
A+XL_S
B+XL_L
y14
b4
b5
b5
y13
z17
c6
c15
c12
z16
z15
c7
z12
z7
z6
z4
y6
z7
y8
ANVAKPGLVDDFEK+XL_S
A_b_ion
A_y_ion
m/z
400
600
800
1000
1200
1400
2000
6000
ANVAKPGLVDDFEK+XL_L
IMNVIGEPIDERGPIKTK+XL_S
B_b_ion
B_y_ion
y13++
y14++
y16++
4000
8000
14000
IMNVIGEPIDERGPIKTK+XL_L
y11+
y8+
y13+
1000
3000
1500
2000
